# PGD Synthase and PGD**_2_** in Immune Resposne

**DOI:** 10.1155/2012/503128

**Published:** 2012-06-25

**Authors:** Myungsoo Joo, Ruxana T. Sadikot

**Affiliations:** ^1^Division of Applied Medicine, School of Korean Medicine, Pusan National University, Yangsan 627-870, Republic of Korea; ^2^Department of Veterans Affairs, Jesse Brown VA Hospital, Chicago, IL 60612, USA; ^3^Section of Pulmonary, Critical Care, Sleep and Allergy, University of Illinois at Chicago, M/C 719, Chicago, IL 60612, USA

## Abstract

PGD_2_ is formed from arachidonic acid by successive enzyme reactions: oxygenation of arachidonic acid to PGH_2_, a common precursor of various prostanoids, catalyzed by cyclooxygenase, and isomerization of PGH_2_ to PGD_2_ by PGD synthases (PGDSs). PGD_2_ can be either pro- or anti-inflammatory depending on disease process and etiology. The anti-inflammatory and immunomodulatory attributes of PGDS/PGD_2_ provide opportunities for development of novel therapeutic approaches for resistant infections and refractory inflammatory diseases. This paper highlights the role of PGD synthases and PGD2 in immune inflammatory response.

## 1. Introduction

Prostaglandins (PG) are a family of structurally related eicosanoids that have regulatory roles in normal physiological as well as pathological contexts [1]. Cyclooxygenase enzymes catalyze the conversion of arachidonic acid to PGH_2_, which is converted to other prostanoid species including PGD_2_, PGE_2_, PGF_2*α*_, prostacyclin (PGI_2_), and thromboxane (TX) A_2_ by the action of specific synthases [1–3].

 The synthesis of PGD_2_ from its precursor PGH_2_ is catalyzed by two PGD synthases (PGDSs) [4]. Prostaglandin D2 (PGD2) is involved in a wide variety of neurophysiological functions, such as regulation of body temperature, hormone release, modulation of odor and pain responses, and regulation of the sleep-wake cycle in mammals. PGD_2_ is further dehydrated to produce PGJ_2_, Δ^12^-PGJ_2_, and 15-deoxy-Δ^12,14^-PGJ_2_. PGD_2_ acts through two receptors (DP1 and DP2 CRTH2), whereas 15d-PGJ_2_ can activate peroxisome proliferator-activated receptors or inhibit a range of proinflammatory signaling pathways, including NF-*κ*B [1, 2, 5].

 The importance of the role of PGD_2_ in the pathogenesis and resolution of inflammation and innate immune response is increasingly recognized [6, 7]. However, the effect of PGD_2_ on inflammation is complex because PGD_2_ either promotes or suppresses inflammation depending on the inflammatory milieu. This is further complicated by the fact that PGD_2_ undergoes nonenzymatic processes to generate 15d-PGJ_2_, an anti-inflammatory lipid. Therefore, the net effect may depend on the rate of production of distal products of PGD_2_ depending upon the disease process. Here, we review the biology and role of PGD synthases and PGD_2_ in inflammation and host immune response.

## 2. PGD Synthases

The arachidonate cyclooxygenase pathway can generate PGD_2_ by the functional linkage of a series of isoformic enzymes corresponding to phospholipase A_2_, cyclooxygenase and PGDS. Prostanoid formation occurs when cyclooxygenase oxygenates arachidonate converting it to PGG_2_, which is then reduced to PGH_2_. PGH_2_, in turn, is converted to five primary active metabolites, PGD_2_, PGE_2_, PGF_2*α*_, PGI_2_, or thromboxane A_2_ via distinct synthases such as PGD synthase and PGE synthase [1, 2]. Two PGD synthases have been identified, lipocalin (L-PGDS) and hematopoietic (H-PGDS) [4, 8–10]. L-PGDS and H-PGDS are quite different from each other biochemically in terms of their amino acid sequence, tertiary structure, evolutional origin, chromosomal and cellular localization, and tissue distribution and immunologically in terms of their functional relevance [10, 11].

## 3. Hematopoietic PGD Synthase

Hematopoietic PGD synthase (H-PGDS) was previously known as the spleen-type PGDS [9, 12] or glutathione- (GSH-) requiring enzyme for the production of PGD_2_ in the peripheral tissues [12, 13]. H-PGDS is characterized as a member of Sigma class of glutathione *S*-transferase (GST) gene family [14] that catalyze the conjugation of GSH to an electrophilic substrate. The enzyme is a homodimer and folds like other glutathione transferases. H-PGDS is localized in antigen-presenting cells and mast cells of a variety of tissues and is involved in the activation and differentiation of mast cells. It is also expressed in dendritic cells, Langerhans, and megakaryoblasts [15]. H-PGDS isomerizes PGH_2_ to PGD_2_ selectively and effectively, whereas other GST isozymes catalyze the conversion of PGH_2_ nonselectively to produce PGD_2_, PGE_2_, and PGF_2*α*_. The high specificity of H-PGDS for the production of PGD_2_ is attributed to the unique architecture of the catalytic pocket. The deep and wide catalytic cavity of H-PGDS is striking in comparison with the narrow shallow cavities of other GSTs. The unique 3 D architecture of the cleft leads to the putative substrate binding mode and its catalytic mechanism, responsible for the specific isomerization from PGH_2_ to PGD_2_ [14].

H-PGDS contributes to the production of the D and J series of prostanoids in the immune system and is involved in allergic inflammatory response. Since H-PGDS is present in mast cells, Th2 cells, and other leukocytes, it is thought to be responsible for the bulk of PGD_2_ production during allergic responses [16, 17]. In mouse models of asthma and allergic disease, H-PGDS has a substantial proinflammatory effect, regulating many hallmark characteristics including eosinophilia, airway hyperreactivity, mucus production, and Th2 cytokine levels. Inhibitors of H-PGDS have shown to be protective in mouse models of allergic airway inflammation [18]. The compound, HQL-79, is characterized as a specific inhibitor of human H-PGDS and has shown to exhibit a therapeutic effect when used in animal models of allergic disease and neuroinflammation [19]. Thus, selective inhibitors of H-PGDS may prove to be more useful to suppress allergic and inflammatory reactions rather than COX-1 or COX-2 inhibitors, such as aspirin, indomethacin, and coxibs because these COX inhibitors suppress the production of all prostaglandins in comparison to H-PGDS inhibitors [20–23].

While H-PGDS is proinflammatory in allergic airway diseases, H-PGDS has shown to be protective in other models of inflammation. Trivedi et al. showed that H-PGDS negatively regulates the severity and duration of delayed type hypersensitivity responses. Their data suggests that contrary to the role of H-PGDS in driving Th2-like responses in models of asthma, HPGDS may act as an internal braking signal essential for bringing about the resolution of Th1-driven delayed type hypersensitivity reactions [24]. Rajakariar et al. using H-PGDS knockout mice showed that H-PGDS synthesizes 15d-PGJ_2_ during mammalian defense responses and together with PGD_2_, acting through the DP1 receptor, plays a central role in controlling the onset of acute inflammation and its resolution by balancing pro- versus anti-inflammatory cytokines. These data highlight the anti-inflammatory and proresolution properties of cyclopentanone prostaglandins, PGD2 and DP1 receptors [25].

## 4. Lipocalin-Type PGD Synthase

Lipocalin-type PGDS is GSH independent and is identical to beta trace protein, which was discovered in 1961 as a major protein of human cerebrospinal fluid [26–28]. Because it resembles lipophilic ligand carrier proteins it was named lipocalin-type PGD synthase. L-PGDS is a bifunctional protein, acting as a PGD_2_-producing enzyme as well as an intercellular transporter of retinoids or other lipophilic molecules [29]. It is the only enzyme among the members of the lipocalin gene family that binds small lipophilic substances like retinoic acid, bilirubin, and ganglioside. Structurally it is a monomer with a *β*-barrel structure and a hydrophobic pocket and was initially identified as responsible for PGD_2_ production in the brain [10, 19, 30]. Since then it has been shown that L-PGDS is also expressed in other tissues including the heart, kidneys [31, 32], and lungs [33, 34].

L-PGDS is secreted into various body fluids, such as CSF, plasma, seminal plasma, and urine, and functions as both a PGD_2_-producing enzyme and an extracellular transporter of various lipophilic substances. The L-PGDS/*β*-trace concentration in human serum fluctuates with circadian rhythmicity and exhibits a nocturnal increase and is best known because of its ability to induce sleep. The role of L-PGDS in several metabolic functions has since been identified. Deletion of L-PGDS leads to accelerated glucose intolerance and induces obesity [35], nephropathy, and aortic thickening [36, 37]. In animal models of ischemia lack of L-PGDS confers susceptibility to injury in brain and heart [31, 32, 38]. L-PGDS also has an inhibitory effect on progression of lung, ovarian, and colorectal cancer and some forms of leukemia [39]. Thus, it is evident that L-PGDS has several key regulatory roles that extend beyond its function in the brain.

Similar to H-PGDS in models of allergic inflammation L-PGDS has shown to be proinflammatory. Fujitani et al. reported that L-PGDS transgenic mice exhibit strong allergic lung responses and eosinophilia [40] with enhanced allergic airway inflammation. In a model of chronic allergic dermatitis blockade of L-PGDS with an inhibitor led to significant attenuation of inflammatory response [41], which was also confirmed in CRTH2 knockout mice. The proinflammatory role of L-PGDS has also been suggested in human ulcerative colitis. Hokari et al. showed that the level of L-PGDS mRNA expression is increased in UC patients in parallel with disease activity [42]. In a diabetic rat model Ogawa et al. showed that urinary excretion of L-PGDS increased preceding diabetic nephropathy [43] and the levels of L-PGDS could predict the progression of renal injury [44]. These findings have been independently confirmed by other investigators [45]. L-PGDS in the urine is being investigated as a diagnostic biomarker of acute kidney injury and inflammation associated with diabetes, hypertension, and drug-induced nephropathy [46]. Because L-PGDS has a smaller molecular weight than serum albumin it may be expected to appear in the urine even before albuminuria and hence prove as a more sensitive marker for early detection of renal injury.

We have studied the role of L-PGDS in LPS-induced inflammation and shown that L-PGDS is induced *in vitro* in macrophages [33, 34] and *in vivo* in the lung in response to LPS and *P. aeruginosa* [33]. Our study showed that H-PGDS was constitutively expressed *in vitro* in macrophages whereas L-PGDS is induced in a time-dependent manner in response to LPS or PA103. Similarly *in vivo* studies in mice showed that the expression of L-PGD synthase was induced in response to LPS and PA103 [33]. However, the immunomodulatory effects of L-PGDS are less well studied. In a mouse model of *P. aeruginosa* infection we have shown that L-PGDS^−/−^ mice have impaired host defenses whereas overexpression of L-PGDS is protective in *P. aeruginosa*-induced pneumonia suggesting a pivotal role for L-PGDS in innate immune response [33]. These studies suggest an important role of L-PGDS in immunomodulation.

## 5. Prostaglandin D_2_

PGs are a group of 20-carbon polyunsaturated fatty acids containing a unique 5-carbon ring structure. Prostaglandins are all produced from arachidonic acid (C20:4 fatty acid) via their common intermediate, PGH_2_, and are a family of structurally related eicosanoids that not only have an important role in homeostasis but also contribute to the pathology of numerous inflammatory diseases. Each prostanoid is then produced from PGH_2_ by its specific terminal PG synthase such as PGD synthase in the case of PGD_2_ [13]. PGD_2_ is an acidic lipid mediator derived from arachidonic acid by sequential action of cyclooxygenase and PGD_2_ synthases. Both H- and L-PGD synthase enzymes may form PGD_2_ *in vitro,* but it is not fully understood which PGDS enzyme predominates under varied conditions *in vivo*.

PGD_2_ for a long time was considered as a minor and biologically inactive prostaglandin. In the 1980s, however, PGD_2_ was found to be the most abundant prostaglandin in the brains of rats [52] and other mammals including humans [53], thus suggesting that it may have an important function in the central nervous system (CNS). The physiological functions of PGD_2_ have now been extensively defined and include regulation of sleep and body temperature, olfactory function, hormone release, and nociception in the central nervous system. PGD_2_ also prevents platelet aggregation and induces vasodilation and bronchoconstriction. It is released from mast cells as an allergic and inflammatory mediator [54] and is responsible for the symptoms in mastocytosis patients, such as flushing, diarrhea, tachycardia, dyspnea, and deep sleep [55].

PGD_2_ is further converted to 9*α*, 11*β*-PGF_2_, a stereoisomer of PGF_2*α*_, which exerts various pharmacological actions different from those induced by PGF_2*α*_. PGD_2_ is also readily dehydrated *in vitro* and *in vivo* [56] to produce prostaglandins of the J series, such as PGJ_2_, Δ^12^-PGJ_2_, and 15-deoxy-Δ^12,14^-PGJ_2_. 15-Deoxy-Δ^12,14^-PGJ_2_ has been identified as an endogenous ligand for a nuclear receptor (peroxisome proliferator-activated receptor *γ*), and it promotes adipocyte differentiation [5, 6].

Prostaglandin D_2_ may exert proinflammatory or anti-inflammatory effects in different biologic systems. PGD_ 2_ has been implicated in the initiation and progression of inflammation. In mouse models of asthma and allergic disease, PGD_2_ has a substantial proinflammatory effect, regulating many hallmark characteristics including eosinophilia, airway hyperreactivity, mucus production, and Th2 cytokine levels [40, 47]. Moreover, inhibition of PGD_ 2_ synthesis and PGD_ 2_ signaling blockade has a suppressive effect on neuroinflammation in mouse models of Krabbe's disease [48]. The injection of PGD_ 2_ into the skin has been shown to result in erythema, edema, induration, and leukocyte infiltration [49]. PGD_ 2_ and other vasodilator prostaglandins may also contribute to inflammation by increasing local blood flow.

 In contrast to these proinflammatory effects, PGD_ 2_ and its cyclopentenone prostaglandin derivatives also have anti-inflammatory properties, with functions in resolution of inflammation. There is considerable interest in the importance of PGD_2_ and its distal products in the mediation and resolution of inflammation [3, 57]. Gilroy et al. showed that in a model of experimental pleuritis PGD_2_ significantly attenuated inflammation [3]. Similarly in a model of experimental colitis COX-2-derived PGD_2_, acting via the DP receptor, was shown to attenuate neutrophilic infiltration into colonic mucosa [50]. In a human model of an acute inflammatory response induced by administration of LPS, which evokes transient flu-like symptoms with pyrexia and a hemodynamic response, Song et al. have shown that tetranor PGDM increases markedly during this response and that PGJ_2_ has antipyretic effects [58]. These data strongly support the anti-inflammatory effects of PGD_2_.

 Although several studies have investigated the role of PGD_ 2_ in inflammation, the role of PGD_2_ in host immune response has been scantly studied. An earlier study showed that PGD_2_ concentration, but not the PGE_2_ or IL-1*β* concentrations, is elevated in a time-dependent manner in the CSF of patients with African sleeping sickness, caused by *Trypanosoma brucei* [59]. These investigators have also shown that mouse astrocytes and fibroblasts in culture induce the production of PGD_2_ in response to *T. brucei* [60]. Although the production of PGD2 was increased *in vitro,* the functional effects of PGD2 in this setting remain unclear. In a recent investigation Zhao et al. showed that an age-related increase in PGD_2_ in mice led to diminished respiratory dendritic cell migration resulting in defects in virus-specific T-cell responses *in vivo*. They further showed that administration of PGD_2_ antagonist reversed this defect resulting in migration of dendritic cells with enhancement of T-cell antivirus response with increased clearance and survival [51]. These data suggest that similar to allergic airway disease PGD_2_ may have immunosuppressive effects in viral infections.

In a mouse model of *P. aeruginosa* lung infection we have shown that inhibition of COX-2 improves survival in a lethal model of *P. aeruginosa* infection [61]. The bacterial clearance of *P. aeruginosa* was enhanced in COX-2 knockout mice whereas transgenic mice that overexpress COX-2 have an impaired bacterial clearance from the lungs [62]. Our study showed that the immunomodulatory effects of inhibition of COX-2 are related to inhibition of PGE_2_. We also examined the effects of administration of PGD_2_ in a model of *P. aeruginosa* lung infection. Mice that were given intratracheal PGD_ 2_ showed an enhanced clearance of *P. aeruginosa* from the lungs [33]. These results were in agreement with our studies from L-PGDS knockout and L-PGDS overexpressing mice [33]. Recently we have investigated the mechanisms by which PGD_2_ may exhibit immunomodulatory effects. We have shown that PGD_2_ inhibits a key proinflammatory immunoglobulin cell surface receptor TREM-1 *in vitro* in macrophages [63]. Furthermore, we have shown that PGD_2_ induces the expression of Nrf2 in a DP1 receptor-dependent manner [63]. These studies provide a new paradigm and highlight a key regulatory role of PGD_2_ in innate immune response to bacterial infections.

## 6. Conclusions

The role of PGDS/PGD_ 2_ in regulating inflammation in a variety of organ systems and disease process is burgeoning. The inflammatory response protects the body against infection and injury but can itself become dysregulated with deleterious consequences to the host. It is now evident that endogenous biochemical pathways such as PGDS/PGD_ 2_ get activated during defense reactions. The effect of PGDS/PGD_2_ on inflammation is complex because they can either promote or suppresses inflammation depending on the inflammatory milieu.  Table 1 provides a summary of the models of different effects of PGDS/PGD_2_. Interdiction of L-PGDS, PGD_2_, and DP receptors provides novel therapeutic approaches to modulate inflammation and innate immune responses.

## Figures and Tables

**Table 1:** Summary of PGDS and PGD_2_ effects in models of inflammation.

	Model	Reference
*H-PGDS*		
Proinflammatory	Allergic airway inflammation (mouse model)	[18]
Anti-inflammatory	Delayed type hypersensitivity (mouse model)	[24, 25]

*L-PGDS *		
Pro-inflammatory	Allergic airway inflammation (mouse model)	[40]
	Chronic allergic dermatitis (mouse model)	[41]
	Human ulcerative colitis	[42]
	Diabetic nephropathy (mouse Model)	[43–45]
Anti-inflammatory	LPS-induced lung inflammation (mouse model)	[33]
Immunoprotective	*P. aeruginosa* lung inflammation (mouse model)	[33]

*PGD2 *		
Pro-inflammatory	Asthma and allergic airway inflammation (mouse model)	[40, 47]
	Neuroinflammation/Krabbe's disease (mouse Model)	[48]
	Skin inflammation (mouse model)	[49]
Anti-inflammatory	Mouse model of pleuritis	[3]
	Mouse model of colitis	[50]
Immunosuppressive	Viral infection (mouse Model)	[51]
